# ENHANCING SUPPORT SYSTEMS FOR OLDER ADULTS’ HEALTH AND WELL-BEING: A HEALTH EQUITY APPROACH

**DOI:** 10.1093/geroni/igae098.0058

**Published:** 2024-12-31

**Authors:** Ann Nguyen, Katrina Ellis

**Affiliations:** Chair; Co-Chair

## Abstract

This symposium examines psychosocial factors that shape the health and well-being of older adults using a health equity lens. The first paper examines the interplay between racial discrimination, social relationships and cellular aging among older African Americans. The findings from latent class analysis demonstrate significant heterogeneity in experiences of discrimination, social relationships, and susceptibility to molecular stress among African Americans. The second paper investigates protective factors and adverse outcomes associated with elder abuse among persons living with dementia (PLWD) using a systematic review methodology. The results identify several caregiver-related factors that protected against elder abuse and adverse outcomes related to medical conditions and medication adherence. The third paper reports the findings for the effect of urbanicity of residence on the health of caregivers of PLWD using data from the 2020-2022 Behavioral Risk Factor Surveillance Systems surveys. This analysis indicates that caregivers who lived in rural areas were more likely to self-report poor physical health than caregivers who lived in urban areas. The fourth paper examines barriers for caregivers to engage in fall risk management from the perspective of health professionals who work with PLWD and their caregivers using semi-structured, qualitative interview data. Thematic analysis identifies the several themes related to barriers. Overall, these papers will provide important insights into modifiable psychosocial factors that influence health and well-being. Modifiable psychosocial factors have broad translational importance in promoting health equity. The findings will be discussed with attention to important socio-cultural and contextual factors and highlight implications for gerontological practice and research.

